# A systemic study of indoxacarb resistance in *Spodoptera litura* revealed complex expression profiles and regulatory mechanism

**DOI:** 10.1038/s41598-019-51234-5

**Published:** 2019-10-18

**Authors:** Li Shi, Yao Shi, Ya Zhang, Xiaolan Liao

**Affiliations:** 1 Hunan Provincial Key Laboratory for Biology and Control of Plant Diseases and Insect Pests, College of Plant Protection, Hunan Agricultural University, Changsha, 410128 China; 2 Hunan Provincial Engineering and Technology Research Center for Bio-pesticide and Formulation Processing, Changsha, 410128 China

**Keywords:** Biochemistry, Cell biology, Molecular biology

## Abstract

The tobacco cutworm, *Spodoptera litura*, is an important pest of crop and vegetable plants worldwide, and its resistance to insecticides have quickly developed. However, the resistance mechanisms of this pest are still unclear. In this study, the change in mRNA and miRNA profiles in the susceptible, indoxacarb-resistant and field indoxacarb-resistant strains of *S*. *litura* were characterized. Nine hundred and ten co-up-regulated and 737 co-down-regulated genes were identified in the resistant strains. Further analysis showed that 126 co-differentially expressed genes (co-DEGs) (cytochrome P450, carboxy/cholinesterase, glutathione S-transferase, ATP-binding cassette transporter, UDP-glucuronosyl transferase, aminopeptidase N, sialin, serine protease and cuticle protein) may play important roles in indoxacarb resistance in *S*. *litura*. In addition, a total of 91 known and 52 novel miRNAs were identified, and 10 miRNAs were co-differentially expressed in the resistant strains of *S*. *litura*. Furthermore, 10 co-differentially expressed miRNAs (co-DEmiRNAs) had predicted co-DEGs according to the expected miRNA-mRNA negative regulation pattern and 37 indoxacarb resistance-related co-DEGs were predicted to be the target genes. These results not only broadened our understanding of molecular mechanisms of insecticide resistance by revealing complicated profiles, but also provide important clues for further study on the mechanisms of miRNAs involved in indoxacarb resistance in *S*. *litura*.

## Introduction

The tobacco cutworm, *Spodoptera litura* (Fabricius), is an important polyphagous insect pest that causes widespread economic damage to vegetables and other crops, such as soybean, tomato, sweet potato, groundnut and cotton^1,2^. Over many years, the control of *S*. *litura* has traditionally relied on sprays of various insecticides^3^. Many field populations of *S*. *litura* have developed high resistance to multiple insecticides, including organophosphate, carbamate, pyrethroids and some newer chemistry insecticides such as indoxacarb, abamectin, emamectin benzoate and chlorantraniliprole^3–6^. The effort to control this pest is becoming exceedingly challenging all over the world. Therefore, understanding the mode of action and resistance mechanisms of insecticide are extremely important for us to design effective resistance management strategies. Also, identifying the molecular mechanism responsible for insecticide resistance will provide new opportunities for the development of novel strategies for insect pest control.

Studying insecticide resistance makes it possible to classify pest adaptations into two main mechanisms: degradation of metabolic detoxification with elevating enzyme activities or insecticide target insensitivity due to point mutations^7^. Metabolic resistance has been reported worldwide and mostly associated with an increased level of detoxification enzymes (such as cytochrome P450 (P450), carboxy/cholinesterase (CCE), and glutathione S-transferase (GST)) in insecticide-resistant populations^8^. Functional genomics tools have been used to diagnose the genetic basis of pesticide resistance in arthropods^9^. Such studies showed that insecticide resistance is more complex than thought before, mediated by multigenic systems that involve large parts of the insect genomes. However, due to the large number of *S*. *litura* genes encoding detoxification enzymes, pinpointing those responsible for resistance remains challenging^10^.

MicroRNAs (miRNAs) are endogenous small (about 22 nucleotides) non-coding RNAs that regulate gene expression through binding to the 3′untranslated region (3′UTR) of target mRNA, and causing inhibition of translation or mRNA degradation at post-transcriptional level^11,12^. As a key component in post-transcriptional gene expression regulation, miRNAs play important roles in many physiological processes, such as metabolism, growth, development, behavior and apoptosis^13^. Some research has shown that miRNAs were involved in the formation of insecticide resistance in arthropods. For example, it has been demonstrated that *miR-71* and *miR-278-3p* could regulate the pyrethroid resistance in *Culex pipiens*^14^ and *Culex pipiens pallens*^15^, respectively. *MiR-276* and *miR-3016* were found to be responsible for spirotetramat resistance in *Aphis gossypii*^16^, *Tci-miR-1-3p* was involved in cyflumetofen resistance in *Tetranychus cinnabarinus*^17^. However, in *S*. *litura*, there is still insufficient information regarding the relationship between miRNAs and insecticide resistance.

In this study, we employed the high-throughput sequencing platform-Illumina HiSeq 2500 to complete the mRNA and small RNA (sRNA) sequencing from the fourth-instar larva of susceptible strain (SS), lab indoxacarb-resistant strain (InRS) and field indoxacarb-resistant strain (FInRS) of *S*. *litura*. Then we analyzed the expression profiles of the mRNAs and miRNAs from three strains and predicted the targets of miRNAs. Co-differentially expressed genes (co-DEGs) and co-differentially expressed miRNAs (co-DEmiRNAs) in the two resistant strains were screened, and the expression profiles were then validated by quantitative real-time PCR (qPCR) techniques. We further focused on co-DEGs related to insecticide resistance. Finally, the regulatory relationships between co-DEmiRNAs and insecticide resistance-related co-DEGs were analyzed. The aim of the present study, therefore, was to clarify the indoxacarb resistance mechanisms and the regulatory mechanisms of miRNAs in *S*. *litura*.

## Results

### Toxicity of indoxacarb to *S*. *litura*

The bioassays results of indoxacarb showed that the LC_50_ of SS, InRS and FInRS were 0.64 mg/L, 37.37 mg/L and 25.65 mg/L, respectively. The InRS and FInRS exhibited 58.39-fold and 40.08-fold resistance against indoxacarb compared to the SS, respectively (Table 1).

**Table 1:** The susceptibility of SS, InRS and FInRS to indoxacarb in *S*. *litura*.

Strains	LC_50_ (mg/L) 95% CI	Slpoe (±SE)	χ^2^	RR
SS	0.64 (0.40–1.03)	1.32 ± 0.28	1.52	—
InRS	37.37 (25.84–57.43)	1.60 ± 0.40	0.94	58.39
FInRS	25.65 (19.94–33.97)	2.33 ± 0.51	1.04	40.08

LC_50_, median lethal concentration; RR, Resistance ratio.

### Analysis of mRNA sequencing data

In order to identify the mRNA expression profiles in different strains of *S*. *litura*, a total of three libraries, SS, InRS and FInRS, were constructed and sequenced by Illumina HiSeq 2500 platform. The samples from each strain were sequenced in triplicate. The SS, InRS and FInRS libraries were found to contain 163,943,684, 156,829,302 and 177,060,140 raw reads, respectively. After removing low-quality reads, adaptors and all possible contaminants, 161,116,162, 153,986,650 and 173,406,844 clean reads were obtained, respectively. Among them, 89.94%, 89.81% and 86.89% clean reads were uniquely mapped to the reference genome, respectively (Table 2).

**Table 2:** mRNA sequencing statistics.

Sample name	SS	InRS	FInRS
Raw reads	163943684	156829302	177060140
Clean reads	161116162(100.00%)	153986650(100.00%)	173406844(100.00%)
Total mapped	148530249(92.19%)	141712331(92.03%)	155743368(89.81%)
Uniquely mapped	144914231(89.94%)	138302930(89.81%)	150669830(86.89%)
Reads map to ‘+’	72334209(44.90%)	69065291(44.85%)	75185092(43.36%)
Reads map to ‘−’	72580022(45.05%)	69237639(44.96%)	75484738(43.53%)

“+” and “−” mean refers to sense strands and anti-sense strand, respectively.

**Figure 1:**
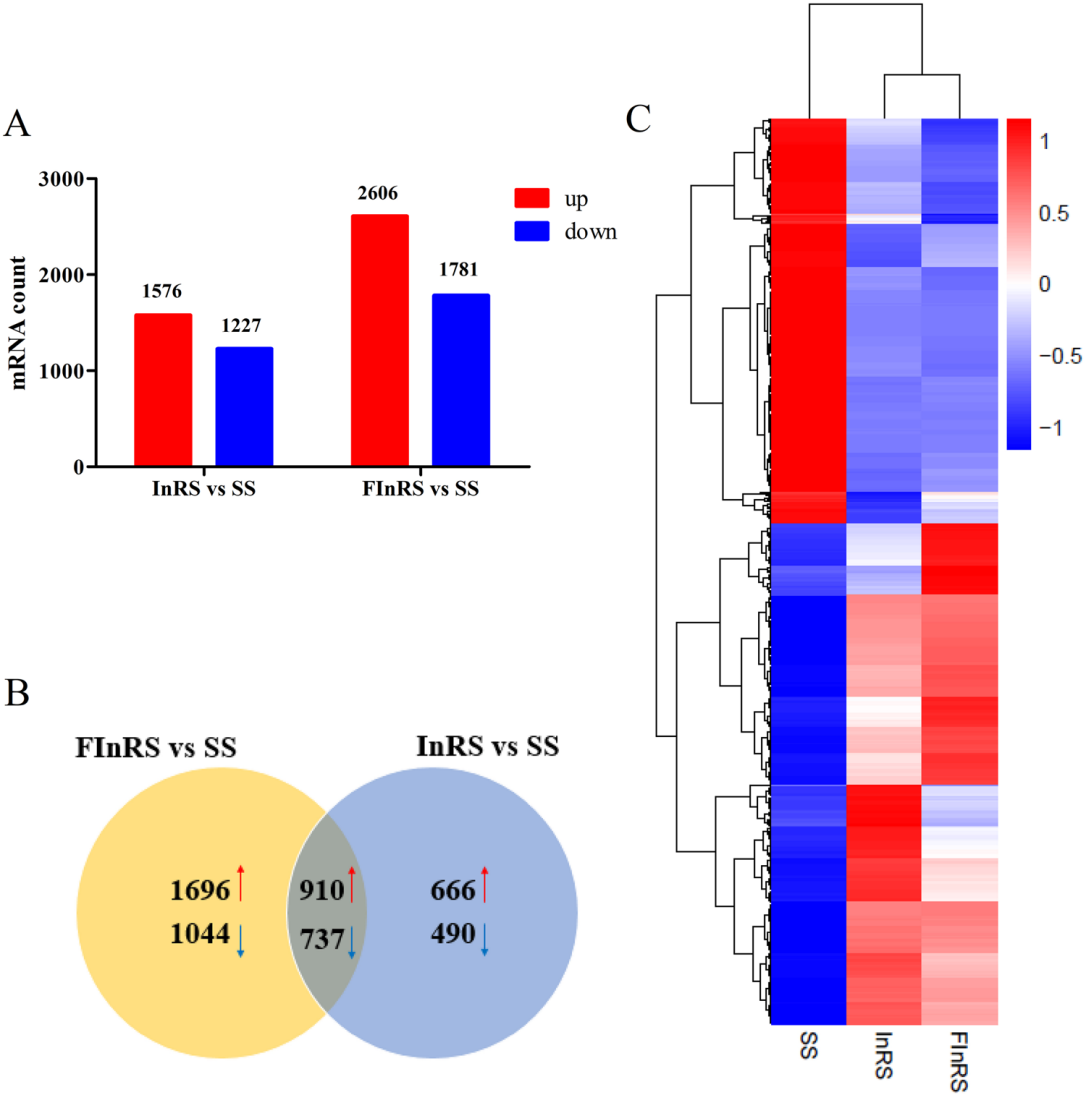
Differentially expressed profiles of mRNAs. (**A**) The number of differentially expressed mRNAs in the SS, InRS and FInRS; (**B**) Venn diagram of differential expression of mRNAs between the InRS *vs*. SS and FInRS *vs*. SS (Red arrows represent up-regulated and blue arrows represent down-regulated); (**C**) Cluster analysis of differentially expressed mRNAs (Parameter setting: data adopt the logarithm: log_10_ and normalization; clustering by row and column).

The DEGs were screened based on DEGSeq 2 analysis taking |log_2_(fold change)| ≥ 1 and *P* < 0.05 as the cut-off between resistant and susceptible strains. Comparing with the SS, 1576 up- and 1227 down-regulated, 2606 up- and 1781 down-regulated genes were identified in the InRS and FInRS of *S*. *litura*, respectively (Fig. 1A). Venn diagram was generated using DEGs and it was depicted that 910 genes were co-up-regulated and 737 genes were co-down-regulated in the InRS and FInRS of *S*. *litura* (Fig. 1B). A cluster heat map was subsequently adopted to show co-DEGs in the InRS and FInRS of *S*. *litura*. co-DEGs were divided into two groups with four clusters, and the gene expression of the InRS is similar to that of the FInRS, but they were differentially expressed compared to the SS (Fig. 1C). Furthermore, GO analysis results showed that these co-DEGs were divided into three ontologies: biological process, cellular component, and molecular function, including 26 GO terms, and the most enriched functions were structural molecule activity, transmembrane transport, extracellular region, cofactor binding and lipid metabolic process (Supplementary Fig. S1).

### Analysis of sRNA sequencing data

In order to clarify the miRNA expression profiles in different strains of *S*. *litura*, sRNA libraries were also constructed and sequenced by the Illumina HiSeq 2500 platform. The samples from each strain were sequenced in triplicate. A total of 27,365,328, 42,891,738 and 42,263,830 raw reads were found in the SS, InRS and FInRS libraries, respectively. Firstly, removing the low-quality reads, adaptors and ploy A or T or G or C, 23,197,151, 38,421,746 and 32,838,871 clean reads were obtained and used for further analysis (Table 3). After discarding the sequences shorter than 18 nt or more than 35 nt, 11,146,105 (82.70%), 17,943,993 (80.93%) and 8,786,448(61.40%) clean reads were uniquely mapped to the reference genome, respectively (Supplementary Table S3), and different RNA categories of matched sRNA were analyzed (Supplementary Table S4).

**Table 3:** Small RNA sequencing statistics.

Sample name	SS	InRS	FInRS
Raw reads	27365328(100.00%)	42891738(100.00%)	42263830(100.00%)
N% > 10%	1371(0.01%)	808(0.002%)	1411(0.003%)
Low quality	414252(1.51%)	654591(1.53%)	657318(1.56%)
5′ adapter contamine	7498(0.03%)	11746(0.03%)	12594(0.03%)
3′ adapter null or insert null	3730728(13.63%)	3776699(8.81%)	8720177(20.63%)
With ployA/T/G/C	14328(0.05%)	26148(0.06%)	33459(0.08%)
Clean reads	23197151(84.77%)	38421746(89.58%)	32838871(77.70%)

**Figure 2:**
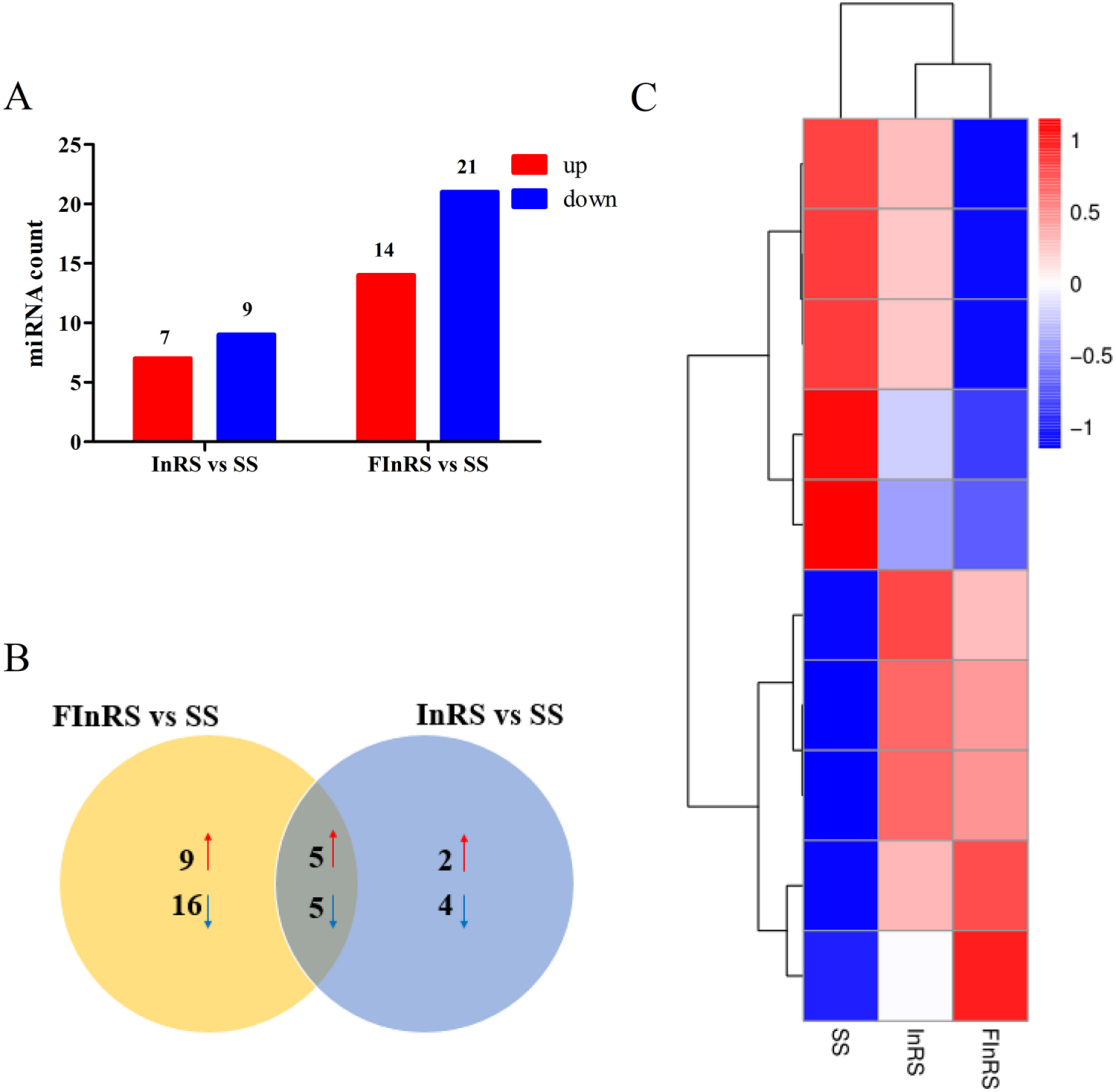
Differentially expressed profiles of miRNAs. (**A**) The number of differentially expressed miRNAs in the SS, InRS and FInRS; (**B**) Venn diagram of differential expression of miRNAs between the InRS *vs*. SS and FInRS *vs*. SS (Red arrows represent up-regulated and blue arrows represent down-regulated); (**C**) Cluster analysis of differentially expressed miRNAs (Parameter setting: data adopt the logarithm: log_10_ and normalization; clustering by row and column).

Through the bioinformatics analysis, 91 known miRNAs and 52 novel miRNAs were identified in the SS, InRS and FInRS of *S*. *litura* (Supplementary Table S5). A total of 7 up- and 9 down-regulated, 14 up- and 21 down-regulated miRNAs were detected in the InRS and FInRS of *S*. *litura*, respectively (Fig. 2A). Venn diagram showed that 5 miRNAs were co-up-regulated and 5 miRNAs were co-down-regulated in the InRS and FInRS of *S*. *litura* (Fig. 2B). The cluster heat map results of co-DEmiRNAs depicted that there are much more differences in miRNAs expressed in the SS, InRS and FInRS of *S*. *litura* (Fig. 2C).

### Validation of expression profiles by qPCR

**Figure 3:**
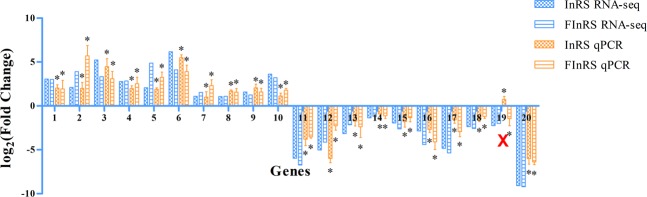
Validation for the expression of selected co-DEGs by qPCR. The qPCR results were analyzed by an independent-sample t-test with a significance level of *P* < 0.05.

**Figure 4:**
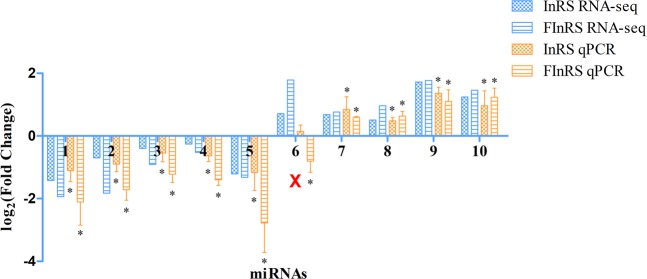
Validation for the expression of co-DEmiRNAs by qPCR. The qPCR results were analyzed by an independent-sample t-test with a significance level of *P* < 0.05.

To validate RNA sequencing results, we used qPCR to investigate the relative expression levels of randomly selecting 20 co-DEGs and all 10 co-DEmiRNAs. The results revealed that 19 of these co-DEGs (95.0%) (Fig. 3) and 9 of these co-DEmiRNAs (90.0%) (Fig. 4) were consistent with RNA sequencing. The qPCR results validated the RNA sequencing results and increased the accuracy and reliability of the differentially expressed genes and miRNAs.

### DEGs Encoding P450, CCE and GST

**Figure 5:**
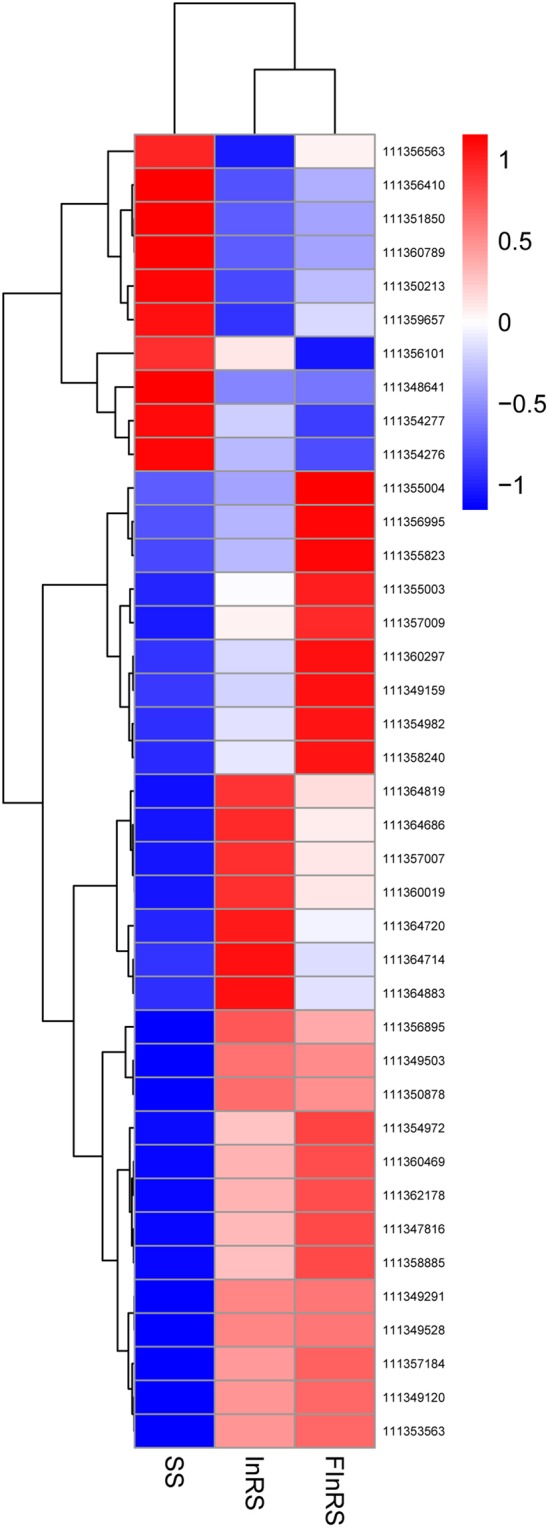
Cluster analysis of differentially expressed detoxification enzyme genes. Detoxification enzyme genes contain P450, CCE and GST; Parameter setting: data adopt the logarithm: log_10_ and normalization; clustering by row and column.

When considering the metabolic enzyme genes potentially involved in resistance, the strong response of the resistant strains against indoxacarb selection through transcription level modifications was confirmed, with several detoxification genes being over-expressed (Fig. 5). Studying P450 genes expression revealed that 24 P450 genes showed significant transcription level variations (19 genes co-up-regulated and 5 genes co-down-regulated) in the InRS and FInRS (Table 4). The differently expressed P450 genes distributed in all P450 clans (clan 2, 3, 4, and M) in the InRS and FInRS. The fold change of these up-regulated P450 genes ranged from 2.11-fold (*SlituP450-003*) to 66.46-fold (*SlituP450-018*) and 2.78-fold (*SlituP450-037*) to 199.98-fold (*SlituP450-085*) in the InRS and FInRS, respectively (Table 4).

**Table 4:** Differential expressions of detoxification enzyme genes in the SS, InRS and FInRS of *S*. *litura*.

Gene ID	Gene name	Clan/Class	Log_2_(Fold Change)	
			InRs	FInRS
**P450**				
111347816	*SlituP450-044*	CYP428A1_CLAN-mit	1.38	1.58
111349291	*SlituP450-063*	CYP341B15_CLAN4	4.06	3.96
111349503	*SlituP450-137*	CYP341B22_CLAN4	2.02	1.72
111349528	*SlituP450-067*	CYP341B_CLAN4	3.06	3.01
111354972	*SlituP450-056*	CYP4S8_CLAN4	1.40	1.73
111354982	*SlituP450-015*	CYP332A1_CLAN3	1.10	2.22
111355003	*SlituP450-086*	CYP6B38_CLAN3	2.11	3.91
111355004	*SlituP450-085*	CYP6B38_CLAN3	2.28	7.64
111355823	*SlituP450-094*	CYP321A10_CLAN3	2.08	6.36
111356895	*SlituP450-018*	CYP339A1_CLAN-mit	6.05	5.34
111356995	*SlituP450-136*	CYP421B1_CLAN4	4.53	7.52
111357007	*SlituP450-132*	CYP340A_CLAN4	3.42	2.47
111357009	*SlituP450-129*	CYP340A_CLAN4	3.53	4.46
111357184	*SlituP450-059*	CYP340K14_CLAN4	4.19	4.41
111358240	*SlituP450-003*	CYP333B3_CLAN-mit	1.08	2.02
111360019	*SlituP450-037*	CYP367A12_CLAN4	2.41	1.48
111360297	*SlituP450-071*	CYP6AE43_CLAN3	1.27	2.81
111360469	*SlituP450-038*	CYP367B11_CLAN4	2.58	2.89
111362178	*SlituP450-119*	CYP324A16_CLAN3	1.76	2.07
111348641	*SlituP450-017*	CYP338A1_CLAN3	−5.96	−6.72
111351850	*SlituP450-005*	CYP18B1_CLAN2	−3.39	−2.14
111354276	*SlituP450-008*	CYP301B1_CLAN-mit	−1.36	−2.13
111354277	*SlituP450-007*	CYP301A1_CLAN-mit	−2.28	−3.99
111360789	*SlituP450-011*	CYP305B1_CLAN2	−5.02	−4.13
**CCE**				
111349120	*SlituCOE090*	Lepidopteran esterase	2.77	2.85
111350878	*SlituCOE093*	Lepidopteran esterase	2.50	2.17
111353563	*SlituCOE050*	Lepidopteran esterase	2.15	2.21
111358885	*SlituCOE009*	α-esterase	1.62	1.82
111364686	*SlituCOE073*	Integument esterase	5.22	3.32
111364720	*SlituCOE111*	Integument esterase	2.96	1.44
111364819	*SlituCOE074*	Integument esterase	3.83	2.28
111350213	*SlituCOE076*	Juvenile hormone esterase	−3.16	−2.15
111356410	*SlituCOE062*	Lepidopteran esterase	−1.36	−1.20
111356563	*SlituCOE067*	Lepidopteran esterase	−3.45	−1.46
111359657	*SlituCOE091*	Lepidopteran esterase	−1.92	−1.21
**GST**				
111349159	*SlituGST20*	ε	2.08	4.88
111364883	*SlituGST38*	ε	6.18	4.11
111356101	*SlituGST42*	σ	−1.37	−6.18

Another important metabolic enzyme related to insecticide resistance is CCE. The number of differently expressed CCE genes was less than the number of P450 genes. Comparing  with the SS, there were 11 CCE genes having differential expression (7 genes co-up-regulated and 4 genes co-down-regulated) (Table 4). Three of the up-regulated CCE genes belonged to lepidopteran esterase, one of these genes belonged to α-esterase and three of these genes belonged to integument esterase (Table 4). The fold change ranged from 3.07-fold (*SlituCOE009*) to 37.20-fold (*SlituCOE073*) and 2.72-fold (*SlituCOE111*) to 10.01-fold (*SlituCOE073*) in the InRS and FInRS, respectively (Table 4).

GST was also an important detoxification enzyme playing crucial roles in insecticide metabolic resistance. In this study, there were 3 GST genes differentially expressed (2 genes co-up-regulated and 1 gene co-down-regulated) in the InRS and FInRS (Table 4). The fold change of these two up-regulated GST genes (*SlituGST20* and *SlituGST38*) were 4.24-, 72.65-fold and 29.47-, 17.30-fold in the InRS and FInRS, respectively, all belonging to class epsilon. (Table 4).

### Other DEGs related to insecticide resistance

There are several other insecticide resistance-related genes that were up-regulated in the InRS and FInRS, such as 10 UDP-glucuronosyl transferase (UGT), 9 ATP-binding cassette (ABC) transporter and 1 aminopeptidase N (APN) genes. These genes were detected to be co-up-regulated in the InRS and FInRS (Supplementary Table S6).

There is also a sialin gene with 2.96-fold and 2.99-fold over-expressed in the InRS and FInRS, respectively (Supplementary Table S6). Sialin belongs to the anion/cation symporter (ACS) family, which is a large subfamily of the major facilitator superfamily (MFS) of transporters. Another family of up-regulated genes associated with insecticide resistance is serine protease (SP). Six SP genes were identified as over-expressed genes, with fold change ranged from 2.27- to 5.75-fold and 3.03- to 13.33-fold in the InRS and FInRS, respectively (Supplementary Table S6). In addition, a number of genes encoded cuticle protein (CP) were over-transcribed in the InRS and FInRS with fold change ranged from 2.97- to 674.37-fold and 3.20- to 872.19-fold among the 52 up-regulated CP genes (Supplementary Table S6).

### miRNAs target genes prediction and correlation analysis of miRNAs-mRNAs

According to the sequence information of the known and novel miRNAs, miRNAs target genes prediction were conducted. As a result, a total of 16,235 target genes were predicted in our study (Supplementary Table S7). Further analysis showed that 746 co-DEGs were predicted as all 10 co-DEmiRNAs target genes (Supplementary Table S8). To explore the potential function of target co-DEGs, GO annotation enrichment analysis was conducted. The result of GO annotation enrichment showed that most of these predicted target co-DEGs were mainly focused on structural molecule activity, structural constituent of cuticle and oxidoreductase activity (Supplementary Fig. S2).

Additional annotation focused on putative insecticide resistance-related target co-DEGs of co-DEmiRNAs. These putative insecticide resistance-related genes include 24 P450, 11 CCE, 3 GST, 12 ABC transporter, 11 UGT, 2 APN, 1 sialin, 8 SP and 54 CP genes (126 co-DEGs (107 co-up-regulated and 19 co-down-regulated), Table 4 and Supplementary Table S6). Considering the expected pattern of negative regulation between miRNA and mRNA, 10 co-DEmiRNAs (5 co-down-regulated and 5 co-up-regulated) were associated with 37 predicted co-DEGs (33 genes co-up-regulated and 4 genes co-down-regulated). Among them, 33 co-up-regulated genes contained 6 P450, 2 CCE, 2 ABC, 2 UGT, 3 SP and 18 CP genes, and 4 co-down-regulated genes included 1 P450, 1 GST, 1 APN and 1 CP genes (Table 5).

**Table 5:** The potential insecticide resistance-related target co-DEGs of the co-DEmiRNAs.

miRNAs	Log_2_(Fold Change)		Insecticide resistance-related target co-DEGs
	InRS	FInRS	
*miR-13b-3p*	−1.42	−1.94	*SlituP450-038*, *SlABCC4-2*, *SlituUGT10*, *SlituCP17*, *SlituCP20*, *SlituCP23*, *SlituCP25*, *SlituCP27*, *SlituCP35*, *SlituCP39*, *SlituCP43*, *SlituCP48*, *SlituCP46*
*miR-2c-3p*	−0.70	−1.83	*SlituP450-038*, *SlABCC4-2*, *SlituUGT10*, *SlituCP17*, *SlituCP20*, *SlituCP23*, *SlituCP25*, *SlituCP27*, *SlituCP35*, *SlituCP39*, *SlituCP43*, *SlituCP48*, *SlituCP46*
*novel_3*	−0.26	−0.52	*SlituUGT01*, *SlituCP51*, *SlituCP28*
*novel_15*	−0.40	−0.91	*SlituP450-044*, *SlituP450-136*, *SlituP450-132*, *SlituP450-071*, *SlituP450-003*, *SlituCOE074*, *SlituCOE050*, *SlABCC4-6*, *SlituSP05*, *SlituSP02*, *SlituSP06*, *SlituCP02*, *SlituCP03*, *SlituCP04*, *SlituCP14*, *SlituCP21*, *SlituCP47*, *SlituCP48*
*novel_42*	−1.21	−1.32	*SlituCOE074*
*miR-277-3p*	1.72	1.77	*SlituP450-005*, *SlituCP53*
*miR-2766-3p*	0.51	0.96	*SlituCP53*
*miR-10-5p*	0.71	1.79	*SlituCP53*
*miR-14-3p*	0.68	0.76	*SlituCP53*
*novel_30*	1.24	1.46	*SlituGST42*, *SlituAPN18*

## Discussion

Indoxacarb is a novel oxadiazine insecticide which has good field activity against a number of lepidopteran pests, as well as certain homopteran and coleopteran pests^18^. Indoxacarb can be metabolized by insect esterases or amidases to a *N-*decarbomethoxylated metabolite (DCJW), which is a more active sodium channel blocker than indoxacarb, resulting in paralysis and death of target pest species^19,20^. Because of its safety to mammals and non-target organisms, favorable environmental and residue properties, broad spectrum and rapid inhibition of insect feeding making indoxacarb a powerful new insecticide for crop protection^18^. However, due to intensive use of indoxacarb, many studies have shown that several insects have developed resistance to indoxacarb in recent years, including *Choristoneura rosaceana*^21^, *Musca domestica*^22^, *Plutella xylostella*^23,24^, *Spodoptera exigua*^25^ and *Helicoverpa armigera*^26^. In *S*. *litura*, field populations of *S*. *litura* collected from various areas of Pakistani during 2004–2006 and 2009–2011 developed indoxacarb resistance from 3- to 95-fold^3^ and 7- to 87-fold^27^, respectively. Monitoring insecticide resistance of *S*. *litura* showed that the field populations of Hunan Province had produced 1.92- to 53.4-fold resistance to indoxacarb during 2010–2012 and 2014–2016^6,28^, and the field populations of Sichuan Province had developed 2.2- to 31.0-fold resistance to indoxacarb during 2014–2016^29^.

The mechanisms mediating resistance to indoxacarb have been studied in several insects. Some mutations in the sodium channel gene have been shown to confer target site insensitivity to the neurotoxic effects of indoxacarb. Wang *et al*.^29^ identified two point mutations (F1845Y and V1848I) in *P*. *xylostella*, and Gao *et al*.^25^ identified one point mutation (L1014F) in *S*. *exigua*, which have been proven to confer high levels of resistance to indoxacarb. Even though enhanced target site insensitivity is important for the resistance of insects, detoxification enzymes are also important factors for the metabolism of insecticides. Shono *et al*.^22^ showed that P450 was involved in the resistance to indoxacarb in *M*. *domestica*. Sayyed and Wright^30^, and Nehare *et al*.^31^ found that esterase and GST were related to resistance to indoxacarb in *P*. *xylostella*. Gao *et al*.^25^ also suggested that carboxylesterase and GST were major factors leading to indoxacarb resistance in *S*. *exigua*. What is more, it was demonstrated that the increased activities of carboxylesterase and P450 were important in conferring indoxacarb resistance in *S*. *litura*^28^. However, the indoxacarb resistance mechanism of *S*. *litura* remains unclear at molecular level.

Among the elucidated metabolic mechanisms of resistance, the most common ones include enhanced detoxification of insecticide by up-regulated P450, CCE and GST^32^. In the previous research, Cheng *et al*.^10^ have shown that 4 P450 (*SlituP450-074*, *SlituP450-088*, *SlituP450-092* and *SlituP450-098*), 2 CCE (*SlituCOE057* and *SlituCOE058*) and 2 GST (*SlituGST07* and *SlituGST20*) genes are linked to tolerance of imidacloprid through induction by imidacloprid and knockdown experiments in *S*. *litura*. In the current study, 19 P450 genes were up-regulated in the indoxacarb-resistant strains and widely distributed in clan 3 (*SlituP450-015*, *SlituP450-085*, *SlituP450-086*, *SlituP450-094*, *SlituP450-071* and *SlituP450-119*), clan 4 (*SlituP450-037*, *SlituP450-038*, *SlituP450-056*, *SlituP450-059*, *SlituP450-063*, *SlituP450-067*, *SlituP450-129*, *SlituP450-132*, *SlituP450-136* and *SlituP450-137*) and clan M (*SlituP450-003*, *SlituP450-018* and *SlituP450-044*). It has been shown that the members of the clan 2, clan 3, clan 4 and clan M of P450s are commonly connected with environmental response/detoxifying functions against xenobiotics and phytotoxins in most arthropod species^33–35^. Seven CCE genes, including 3 lepidopteran esterases (*SlituCOE050*, *SlituCOE090* and *SlituCOE093*), 1 α-esterase (*SlituCOE009*) and 3 integument esterases (*SlituCOE073*, *SlituCOE074* and *SlituCOE111*), and 2 epsilon GST (*SlituGST20* and *SlituGST38*) genes were also up-regulated in the indoxacarb-resistant strains. In insects, the lepidopteran and α classes CCE, and epsilon classe GST have been shown to be associated with xenobiotic detoxification^10,36^. These results confirmed our prediction that overexpressed P450, CCE and GST genes were responsible for indoxacarb resistance in *S*. *litura*. It’s worth noting that only *SlituGST20* may be involved in both imidacloprid and indoxacarb resistance, indicating that different types of insecticide resistance is mediated by different detoxification genes in *S*. *litura*.

In addition to the well-known detoxification gene families involved in insecticide resistance, we also revealed that other insecticide-related genes had significant higher expression levels in the resistant strains. UGT as biotransformation enzymes, widely distributed within living organisms and viruses, were presumed to originally participate in the detoxification process^37^. Overexpressed *P*. *xylostella UGT2B17*^38^ and *T*. *cinnabarinus UGT201D3*^39^ have been shown to be involved in insecticide resistance. In addition to enzymes related to metabolism and conjugation, a number of transporter families, of which ABC transporters are the best studied, also play an important role in xenobiotic tolerance^40^. Sun *et al*.^41^ suggested that ABC transporters might be involved in resistance to multiple insecticides in *Laodelphax striatellus*. What is more, APN has been shown to function as Cry protein receptor in insects^42^ and might be involved in the response to different classes of xenobiotics in *S*. *litura*^10^. In this case, the over-expression of 10 UGT, 9 ABC transporter and 1 APN genes may also associate with the detoxification process of indoxacarb in *S*. *litura*.

By revealing that several other genes with a broad range of biological functions are similarly affected by insecticides, our results suggest that the ability of *S*. *litura* to better tolerate insecticides might also be the consequence of the induction of other proteins involved in a wide range of functions. The major facilitator superfamily (MFS), belonging to secondary active membrane transporters, can transport a wide range of small solutes (including inorganic ions, sugars, amino acids, and xenobiotics) in response to chemiosmotic ion gradients in humans. The roles of MFS on adapting stress from host plant shift and acaricide exposure were reported in *Tetranychus urticae*^43^. Sialin is a member of the MFS of transporters^44^. In the present study, up-regulation of sialin gene in *S*. *litura* may result in a higher efflux of insecticides out of *S*. *litura* cells and developing of insecticide resistance. Serine protease, secreted by pancreas in mammals, mainly function in two aspects: protein digest and hydrolysis as well as activation of all proenzymes secreted by pancreas^45^, which is important in the cellular and the humoral arm of invertebrate immune response. It had been reported that up-regulated SP genes were related to deltamethrin resistance in *C*. *pipiens pallens*^46^. Intracellular proteases might play a role in protein biosynthesis or modified conformation of enzymes as part of this induction process^47^. This mechanism may involve increased supply of precursor amino acids from proteolytic degradation products to the intracellular pool, prior to synthesis of detoxifying enzymes in *S*. *litura* following insecticide exposure. This hypothesis could be supported by another fact that 6 up-regulated SP genes were found in *S*. *litura* responded to indoxacarb in our study. Many studies on cuticle protein suggest that insecticide resistance could manifest as a slower rate of penetration due to higher protein and lipid content in the cuticle and/or greater sclerotization and was caused by increased cuticle genes expression^48^. The overexpression of so many CP genes in this study indicated that this gene family may be involved in indoxacarb resistance in *S*. *litura*.

A total of 737 genes were down-regulated (44.7% of total co-DEGs) in the resistant strains. This phenomenon is very understandable since all living organisms have limited energy inputs. Reduced expressions in these genes would save some energy for resistant *S*. *litura* since the other 55.3% of total co-DEGs were up-regulated. It is generally recognized that overexpressed genes may play more important roles than down-regulated genes in the insecticide resistance. The decreased expression of some metabolic detoxification genes might result from the responses to various endogenous and exogenous compounds, or path of physiological signals^49^. Many studies have deduced the balance mechanism of up- and down-regulated genes, including an adaptive homeostatic response that protects the cell from the deleterious effects of oxidizing species, such as nitric oxide and arachidonic acid metabolites from catalytic and/or metabolic enzymes^50^. In return, this also could be a pathological response to inflammatory processes and a need for the tissue to utilize its transcriptional machinery and energy for the synthesis of other components involved in insecticide resistance^51^.

Analysis of mRNA through high throughput expression profiling using transcriptome analysis methods has provided considerable advances in understanding the molecular base of resistance in insects^52^. In *M*. *domestica*, 1316 genes were identified as being up-regulated in the multiple insecticide resistant strain in comparison to the susceptible strains by transcriptome analysis, and the majority of these up-regulated genes fell within the structural classification of proteins (SCOP) categories of metabolism, regulation and intracellular processes^53^. A similar whole transcriptome study has been carried out in *Culex quinquefasciatus*. The results showed that 367 genes were found to be up-regulated in the highly permethrin-resistant strain, and all P450 genes were up-regulated by at least twice^54^. Bai *et al*.^55^ also revealed that P450 genes (CYP9) were highly expressed in pesticide-exposed *Cimex lectularius* populations by transcriptome and qPCR analysis. In this study, 910 co-up-regulated and 737 co-down-regulated genes were identified in the indoxacarb-resistant strains through mRNA sequencing, and the roles of 126 detoxification-associated genes (107 co-up-regulated and 19 co-down-regulated) in indoxacarb resistance were systematically analyzed. These results provide clues to the identification of potential detoxification genes involved in indoxacarb resistance in *S*. *litura*. Although the two resistant strains have different genetic background, it can be more reasonably screened to obtain insecticide resistance-related genes that reflect the real situation in the field. A similar approach has been used to study the molecular mechanisms of spirodiclofen resistance in *T*. *urticae*^56^.

To date, more than 30,000 miRNAs have been found in over 100 organisms^57^. In *C*. *pipiens*, 100 known miRNAs and 42 novel miRNAs were identified, and 28 miRNAs were differentially expressed in the susceptible and deltamethrin-resistant strains^14^. Seventy-five known miRNAs and 64 novel miRNAs were also identified in the susceptible and fenpropathrin-resistant strains of *T*. *cinnabarinus*, including 12 differentially expressed miRNAs^17^. In this study, we identified 91 known miRNAs and 52 novel miRNAs in *S*. *litura*, 10 of which were co-differentially expressed in the InRS and FInRS. This data has detected significantly more miRNAs than the early report by Rao *et al*.^13^, in which only 58 miRNAs were identified among different developmental stages in *S*. *litura*. The reasons that we can detect increased numbers of miRNAs in our study compared to Rao *et al*.^13^ were the availability of *S*. *litura* whole genome, the improved methods and the upgraded miRBase database.

In general, miRNAs play important gene-regulatory roles by targeting the mRNAs of protein coding genes and repressing their post-transcriptional properties. In this way, down-regulation of a miRNA indicates increased activity of its target gene. It is an important step to identify the target genes of miRNAs for understanding their roles in gene regulatory networks. Therefore, we analyzed the relationship between indoxacarb resistance-related co-DEGs and co-DEmiRNAs in *S*. *litura*. Among 126 indoxacarb resistance-related co-DEGs, 33 up-regulated co-DEGs were predicted target genes of 5 down-regulated co-DEmiRNAs, 4 down-regulated co-DEGs were predicted target genes of 5 up-regulated co-DEmiRNAs. With the deepening of the study of miRNA function, some miRNAs have been shown to be associated with insecticide resistance by regulating resistance related genes in insects and mites. Hong *et al*.^14^ and Lei *et al*.^15^ indicated that *miR-71* and *miR-278-3p* were involved in pyrethroid resistance by targeting *CYP325BG3* and *CYP6AG11* in *C*. *pipiens* and *C*. *pipiens pallens*, respectively. Zhang *et al*.^17^ demonstrated that *Tci-miR-1-3p* could regulate the cyflumetofen resistance through *TCGSTM4* in *T*. *cinnabarinus*. Ma *et al*.^58^ also indicated that *miR-92a* regulates pyrethroid resistance through its interaction with a cuticular protein gene, *CpCPR4* in *C*. *pipiens pallens*. Thus, our results indicated that co-DEmiRNAs of *S*. *litura* may function in the formation of the indoxacarb resistance through regulating the insecticide resistance-related co-DEGs.

In conclusion, mRNA and sRNA libraries were constructed and sequenced in the SS, InRS and FInRS of *S*. *litura*. A total of 1647 co-DEGs (910 co-up-regulated and 737 co-down-regulated) and 143 miRNAs, including 10 co-DEmiRNAs (5 co-up-regulated and 5 co-down-regulated) were identified in the resistant strains of *S*. *litura*. Our data revealed that indoxacarb selection strongly affected the transcription levels of 126 detoxification-associated genes (P450, GST, CCE, ABC transporter, UGT, APN, sialin, SP and CP) involved in indoxacarb resistance. Further analysis showed that 37 indoxacarb resistance-related co-DEGs were predicted to be the target genes of 10 co-DEmiRNAs, and indicated that these miRNAs may regulate the indoxacarb resistance through these indoxacarb resistance-related genes in *S*. *litura*. Overall, the present study help us better understand the indoxacarb resistance mechanisms and the regulatory mechanisms of miRNAs in *S*. *litura* at the molecular level. Functional analysis of these resistance related genes and verification of the regulatory relationships between these miRNAs and their target genes are needed to further elucidate the indoxacarb resistance mechanisms in *S*. *litura*.

## Methods

### Ethics statement

The laboratory population of *S*. *litura* was obtained from the Institute of Zoology, Beijing, China. The field population of *S*. *litura* was collected from the field crops of Changsha City, Hunan Province, China. There was no specific permission required for these collection activities because this insect is a kind of agriculture-harmful pest and distributes worldwide. We confirm that the field collection did not involve endangered or protected species.

### Insect strains and bioassays

SS: the laboratory population of *S*. *litura* was obtained from the Institute of Zoology, Beijing, China and kept in the laboratory under artificial diet and climate without any insecticide exposure. InRS: the laboratory resistant strain was selected with indoxacarb in the laboratory from the SS. FInRS: the field indoxacarb resistant strain was collected from the field crops of Changsha City, Hunan Province, China. The SS/InRS and FInRS have different genetic background. The rearing conditions were set as: 25 ± 2 °C, 65 ± 5% relative humidity (RH) and a 14 h light (L): 10 h dark (D) photoperiod.

Bioassays were conducted with fourth-instar larvae of *S*. *litura* using the artificial diet dipping method^59^. Briefly, indoxacarb (15%, E.I. DuPont de Nemours and Co., Inc., Wilmington, DE, USA) was dissolved in sterilized water to at least 5 concentrations and the mortality was kept at 20–80%. The artificial diet was cut into an area of 2 cm^2^ and a thickness of 5 mm, and dipped into the indoxacarb solution for 10 s, including sterilized water as control. These artificial diets were air dried at room temperature for 5–10 min. Then, fifteen larvae were placed on each treated artificial diet. Each dose was performed in three replicates. The bioassays were kept at 25 ± 2 °C, 65 ± 5% RH and 14:10(L:D) photoperiod. Mortality was recorded 24 h after exposure for indoxacarb. Larvae were considered dead if they failed to make a coordinated movement when prodded with a brush. LC_50_ values were calculated via probit analysis using PoloPlus software (LeOra Software Inc., Berkeley, CA, USA).

### RNA isolation

Total RNAs were extracted from fourth-instar larvae of SS, InRS and FInRS using TRIzol reagent (Invitrogen, Carlsbad, CA, USA), respectively. RNA degradation and contamination was monitored on 1% agarose gels. RNA purity, concentration and integrity were measured using the NanoPhotometer spectrophotometer (IMPLEN, CA, USA), Qubit RNA Assay Kit in Qubit 2.0 Flurometer (Life Technologies, CA, USA) and the RNA Nano 6000 Assay Kit of the Bioanalyzer 2100 system (Agilent Technologies, CA, USA), respectively.

### mRNA sequencing and data analysis

Qualified total RNAs from three strains (three biological replicates for each strain) were used for preparing sequencing libraries using NEBNext Ultra RNA Library Prep Kit for Illumina (NEB, USA) following manufacturer’s recommendations. The library preparations were sequenced on an Illumina Hiseq 2500 platform and 125 bp paired-end reads were generated at the Novogene Bioinformatics Institute (Beijing, China) after the clustering of the index-coded samples was performed on a cBot Cluster Generation System using TruSeq SR Cluster Kit v3-cBot-HS (Illumia) according to the manufacturer’s instructions. In order to obtain clean reads, the low-quality reads, adaptor sequence, ploy A or T or G or C and duplication sequence were removed from raw data. Then, clean reads were aligned to the reference genome of *S*. *litura*^10^ using Hisat2 v2.0.5^60^.

Gene expression levels were estimated by FPKM (expected number of Fragments Per Kilobase of transcript sequence per Millions base pairs sequenced)^61^. DEGs analysis was performed using the DESeq2 R package (1.16.1)^62^. Genes with an adjusted *P* < 0.05 and |log_2_(fold change)| ≥ 1 were assigned as differentially expressed. Gene Ontology (GO) database^63^ was used to identify functional modules of DEGs (*P* < 0.05).

### sRNA sequencing and data analysis

Qualified total RNAs from three strains (three biological replicates for each strain) were used as input materials for the sRNA libraries. Sequencing libraries were generated using NEBNext Multiplex Small RNA Library Prep Set for Illumina (NEB, USA.) following manufacturer’s recommendations and index codes were added to attribute sequences to each sample. Briefly, total RNAs were ligated to 5′ and 3′ adaptors, then first strand cDNA was synthesized by reverse transcriptase. After PCR amplification of the cDNA, the amplified PCR products within 140–160 bp were separated and purified by a 8% polyacrylamide gel. Libraries quality were assessed on the Agilent Bioanalyzer 2100 system using DNA High Sensitivity Chips. After cluster generation, the library preparations were sequenced on an Illumina Hiseq 2500 platform and 50 bp single-end reads were generated at the Novogene Bioinformatics Institute (Beijing, China). Clean reads were obtained by removing reads containing ‘N’ (an unrecognized base) at 10% or higher, with 5′ adapter contaminants, without 3′ adapter or the insert tag, containing ploy A or T or G or C and low-quality reads. After removing reads with a sequence shorter than 18 nt or more than 35 nt, clean reads were mapped to the reference genome of *S*. *litura* using Bowtie software^64^. Then, the matched sRNAs were compared with the mature miRNAs in miRBase 20.0 (http://www.mirbase.org/) to looking for known miRNA of *S*. *litura*, only perfectly matches were accepted and counted. Next, the remained sRNAs were compared with Repeatmasker and Rfam database (ftp://selab.janelia.org/pub/Rfam) to remove protein-coding genes, repeat sequences, ribosomal RNA (rRNA), small cytoplasmic RNA (scRNA), transfer RNA (tRNA), small nuclear RNA (snRNA) and small nucleolar RNA (snoRNA). At last, the remained sRNAs were used to predict the novel miRNA through the two available software miREvo^65^ and mirdeep2^66^.

DEmiRNAs were performed using the DESeq2^62^. Corrected *P* < 0.05 was set as the threshold for significantly differential expression. The miRNAs expression levels were estimated by TPM (transcript per million)^67^. Predicting the target genes of miRNAs were performed by miRanda^68^ and PITA^69^. In addition, GO analysis was also used to explore the functions of the predicted target genes (*P* < 0.05)^63^.

### qPCR validation of mRNA and miRNA

To verify the reliability of RNA sequencing, twenty DEGs and all 10 DEmiRNAs were selected to validate their expression pattern by qPCR. Total RNAs were reverse transcribed using TransScript One-Step gDNA Removal and cDNA Synthesis SuperMix Kit (TransGen, Beijing, China). Primers of mRMA (Supplementary Table S1) and miRNA (Supplementary Table S2) were designed using Primer 3.0 (http://frodo.wi.mit.edu/) and Primer Premier 5.0 (Premier Biosoft International, Palo Alto, CA, USA). *Arginine kinase* (*AK*) and *U6 small nuclear RNA* (*U6*) were used as stable reference genes for qRT-PCR assays of mRNA and miRNA, respectively^70^. The qPCR reactions were performed on Bio-rad Detection iQ2 System (Bio-rad, Hercules, CA, USA) with 20 μL reaction mixtures containing1 μL of diluted cDNA, 10 μL TransStart Tip Green qPCR SuperMix (TransGen, Beijing, China), and 1 μL of each primer (10 μM). The optimized qPCR protocol used for the amplification was: 94°C for 30 s, followed by 40 cycles of 94 °C for 5 s and 60 °C for 30 s. Melting curve analysis was performed from 60 to 95 °C to ensure consistency and specificity of the amplified product. The qPCR experiments were conducted according to minimum information for publication of quantitative real-time PCR experiments (MIQE) guidelines^71^. The expression levels of mRNA and miRNA were calculated using the 2^−ΔΔCt^ method^72^. Three biological and two technical replicates were performed for each mRNA and miRNA. The qPCR results were analyzed by an independent-sample t-test with a significance level of *P* < 0.05 using SPSS 23.0 software (SPSS, Chicago, IL, USA).

## Supplementary information

Supplementary Information

Supplementary Table S5

Supplementary Table S7

Supplementary Table S8
