# Supplementary material for: A systemic study of indoxacarb resistance in Spodoptera litura revealed complex expression profiles and regulatory mechanism
Source: Sci Rep. 2019 Oct 18;9:14997. doi: 10.1038/s41598-019-51234-5 (PMC6802196; doi:10.1038/s41598-019-51234-5)
Supplement: Supplementary file 1 — Supplementary Information [file 41598_2019_51234_MOESM1_ESM.pdf]

Supplementary Information

A systemic study of indoxacarb resistance in *Spodoptera litura* revealed complex expression profiles and regulatory mechanism

Li Shi[1, 2*], Yao Shi[1, 2], Ya Zhang[1, 2], Xiaolan Liao[1, 2*]

[1] Hunan Provincial Key Laboratory for Biology and Control of Plant Diseases and Insect Pests, College of Plant Protection, Hunan Agricultural University, Changsha 410128, China;

[2] Hunan Provincial Engineering and Technology Research Center for Bio-pesticide and Formulation Processing, Changsha 410128, China.

*Corresponding authors: Li Shi, shiliabc@hunau.edu.cn; Xiaolan Liao, lxllxl423@163.com

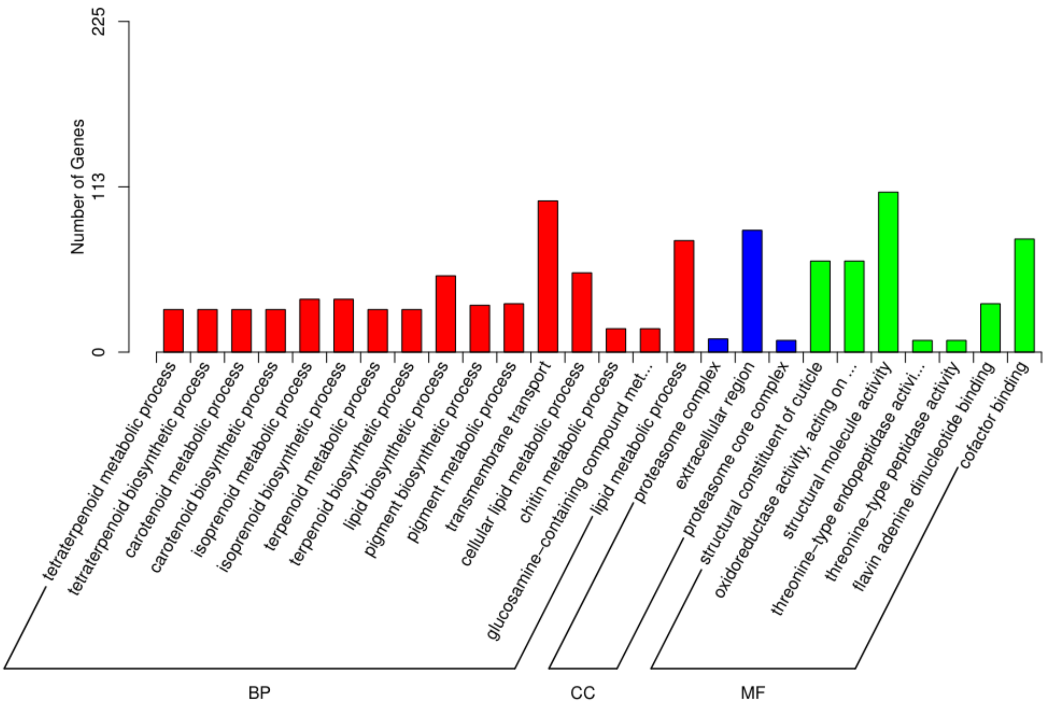

Figure S1. GO classification analysis of co-differentially expressed genes (co-DEGs) in the InRS and FInRS of *S. litura* (*P*<0.05)

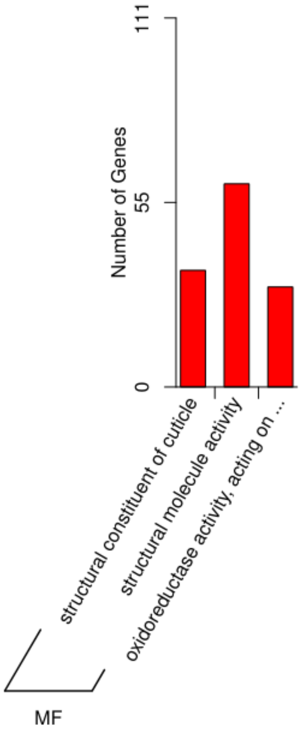

Figure S2. GO classification analysis for predicted co-differentially expressed genes (co-DEGs) of co-differentially expressed miRNAs (co-DEmiRNAs) in the InRS and FInRS of *S. litura* (*P*<0.05)

Table S1. Primers used for qPCR validation of co-differentially expressed genes (co-DEGs)

Number	Gene ID	Forward primers (5'-3')	Reverse primers (5'-3')
1	111349528	CCTGCCTCGACTGAAGTACC	CGGTGTAAAGCCCAGATGTT
2	111355003	GCAAAGAAGGATTGGGACAA	TCTGGAGGAGAGGCTGGATA
3	111364686	TCGCGGTATAGCAATGAGTG	CCTTCACCAAACTCCCAGAA
4	111349120	GGTCTTCAGCATGACCCAAT	ACGGGCTCCACTCCTTCTAT
5	111349159	TATCGCTGGAGACCATGTCA	TCATTTGCACCTGCTTCATC
6	111364883	GAGCTTGTGCTATGCGACAG	CGCGGAATCGAGGATATAAA
7	111348564	ACTCCTTGCCAGCGTACTGT	GTCCCAGCTTCATTCCACAT
8	111349327	GGCTAGCAAGATGGCAGAAC	AAGATGATGATGGGCGAGTC
9	111348017	TGTCTACGAAACCGACAACG	GGAGGAGTGGGGAGGTAATC
10	111358957	GCTCCCAGTGCAGAGAAGAG	ACAGCTGAGGCACCAGTGTA
11	111348641	TCTCAGTCATCGTCGTGCTC	TGGGGTGTTTGGCGTATAGT
12	111360789	GGCATGAGATTGAGCACTGA	CCACACTTCACCGTTAGCAA
13	111350213	AGACTTTCCCTCCCGTCAGT	CGGTTCCTGGATGTTCTCAT
14	111356410	GCCGGTAGGCGATAATACAA	AGTAGCGCTCCTGTCTGAGC
15	111349411	CAGATAGCAGCACTGGTGGA	GTACAGCGGCAGACTGAACA
16	111347869	CGCAAGTAATCCCACCAGTT	TTCAAAAACAACAGCGATGC
17	111349704	CACGGCTCCTAGAAATCCAA	ATGGCGTTGTTCAGCTCTGT
18	111347872	TGGAGGCTCTGTCATCAGTG	TTTGAAGGCGATTTGTACCC
19	111348355	AACGGCACGTACTCCAAAAC	CACACAGGTGGTCATCTTCG
20	111348590	CCCACAAAATAATGCCAAGG	TCAGGTCTTCCAGCTTCGAT
AK	111362331	CTGAAGAAGTACCTTACC	CAATCCAGCAGAGTTGAG

Table S2. Primers used for qPCR validation of co-differentially expressed miRNAs (co-DEmiRNAs)

Number	miRNA	Primer sequences (5'-3')
1	*miR-13b-3p*	RT:CTCAACTGGTGTCGTGGAGTCGGCAATTCAGTTGAGAACTCGTC<br>F: ACACTCCAGCTGGGTATCACAGCCATTTTTG<br>R: TGGTGTCGTGGAGTCG
2	*miR-2c-3p*	RT:CTCAACTGGTGTCGTGGAGTCGGCAATTCAGTTGAGAGTCAACA<br>F: ACACTCCAGCTGGGTATCACAGCCAGCTTT<br>R: TGGTGTCGTGGAGTCG
3	*novel_15*	RT: CTCAACTGGTGTCGTGGAGTCGGCAATTCAGTTGAGCCAGAGCA<br>F: ACACTCCAGCTGGGATTGTACTTCATCAGGT<br>R: TGGTGTCGTGGAGTCG
4	*novel_3*	RT: CTCAACTGGTGTCGTGGAGTCGGCAATTCAGTTGAGACAGCTAT<br>F: ACACTCCAGCTGGGAGGCAAGAAGTCGGCA<br>R: TGGTGTCGTGGAGTCG
5	*novel_42*	RT: CTCAACTGGTGTCGTGGAGTCGGCAATTCAGTTGAGGCAGGCCG<br>F: ACACTCCAGCTGGGAATTGCACCAATCCC<br>R: TGGTGTCGTGGAGTCG
6	*miR-10-5p*	RT: CTCAACTGGTGTCGTGGAGTCGGCAATTCAGTTGAGACAAATTC<br>F: ACACTCCAGCTGGGTACCCTGTAGATCCG<br>R: TGGTGTCGTGGAGTCG
7	*miR-14-3p*	RT: CTCAACTGGTGTCGTGGAGTCGGCAATTCAGTTGAGATAGGAGA<br>F: ACACTCCAGCTGGGTCAGTCTTTTTCTCT<br>R: TGGTGTCGTGGAGTCG
8	*miR-2766-3p*	RT: CTCAACTGGTGTCGTGGAGTCGGCAATTCAGTTGAGACCCACCA<br>F: ACACTCCAGCTGGGTCAGTCTTGTCGAAT<br>R: TGGTGTCGTGGAGTCG
9	*miR-277-3p*	RT: CTCAACTGGTGTCGTGGAGTCGGCAATTCAGTTGAGTGTCGTAC<br>F: ACACTCCAGCTGGGTAAATGCACTATCTGG<br>R: TGGTGTCGTGGAGTCG
10	*novel_30*	RT: CTCAACTGGTGTCGTGGAGTCGGCAATTCAGTTGAGCTCCTGCG<br>F: ACACTCCAGCTGGGCTAAGTACTAGTGCC<br>R: TGGTGTCGTGGAGTCG
U6	-	F: ACCCTCTGTTAGGGGCTGTC<br>R: GGCTCCGTGATTCACACAAT

Table S3. Mapping information statistics with reference genome

Sample name	SS	InRS	FInRS
Total reads	13477246(100.00%)	22172212(100.00%)	14310148(100.00%)
Mapped reads	11146105(82.70%)	17943993(80.93%)	8786448(61.40%)
"+" Mapped reads	6066660(45.01%)	9224711(41.60%)	4675622(32.67%)
"-" Mapped reads	5079445(37.69%)	8719282(39.33%)	4110826(28.73%)

"+" and "-" mean refers to sense strands and anti-sense strand, respectively.

Table S4. Small RNA category annotation statistics

Sample name	SS	InRS	FInRS
Total sRNA	11146105(100.00%)	17943993(100.00%)	8451129(100.00%)
known_miRNA	54899(0.49%)	95276(0.53%)	718386(8.50%)
rRNA	86048(0.77%)	287025(1.60%)	258765(3.06%)
tRNA	4135129(37.10%)	5779681(32.21%)	118346(1.40%)
snRNA	22786(0.20%)	48560(0.27%)	6738(0.08%)
snoRNA	717(0.01%)	2052(0.01%)	347(0.004%)
repeat	36563(0.33%)	69059(0.38%)	36078(0.43%)
novel_miRNA	85657(0.77%)	112463(0.63%)	771093(9.12%)
exon:+	660523(5.93%)	1260014(7.02%)	583759(6.91%)
exon:-	60292(0.54%)	60695(0.34%)	805913(9.54%)
intron:+	480704(4.31%)	922330(5.14%)	961058(11.37%)
intron:-	134146(1.20%)	349886(1.95%)	426677(5.05%)
Unannotated sRNA	5388641(48.35%)	8956952(49.92%)	3763969(44.54%)

rRNA, ribosome RNA; scRNA, small cytoplasmic RNA; snRNA, small nuclear RNA; snoRNA, small nucleolar RNA; tRNA, transfer RNA.

Table S6. Differential expressions of other insecticide-related genes in the SS, InRS and FInRS of *S. litura*

Gene ID	Annotation	Gene name	Log_2(Fold Change)	
			InRs	FInRS
**ATP-binding cassette(ABC)**				
111348564	ATP-binding cassette sub-family G member 4-like	*SlituABCG1-2*	1.10	1.55
111349327	ATP-binding cassette sub-family B member 7	*SlituABCB7*	1.06	1.08
111357088	ABC transporter G family member 20	*SlituABCH1*	1.29	5.05
111360794	multidrug resistance protein homolog 49-like	*SlituABCB3-1*	1.56	1.20
111358751	multidrug resistance-associated protein 4-like	*SlituABCC4-2*	1.70	1.95
111353299	multidrug resistance-associated protein 4-like	*SlituABCC4-1*	1.36	1.22
111361102	multidrug resistance protein homolog 49-like	*SlituABCB3-2*	1.59	2.29
111358857	multidrug resistance-associated protein 4-like	*SlABCC4-6*	1.33	3.06
111353692	multidrug resistance-associated protein 4-like	*SlituABCC3*	1.07	1.40
111349411	ATP-binding cassette sub-family G member 5	*SlituABCG5*	-1.94	-2.61
111348325	ATP-binding cassette sub-family G member 1-like	*SlituABCG1-3*	-2.34	-1.78
111349537	ATP-binding cassette sub-family G member 8	*SlituABCG8*	-1.27	-2.27
**UDP-glucuronosyltransferase (UGT)**				
111348859	UDP-glucuronosyltransferase 2B15-like	*SlituUGT01*	1.23	2.10
111348863	UDP-glucuronosyltransferase 2B15-like	*SlituUGT02*	2.71	4.93
111349001	UDP-glucuronosyltransferase 2B10-like	*SlituUGT03*	1.08	1.06
111351433	UDP-glucuronosyltransferase 2B2-like	*SlituUGT04*	2.11	1.61
111355746	UDP-glucuronosyltransferase 2B15-like	*SlituUGT05*	1.17	3.14
111364538	UDP-glucuronosyltransferase 2B15-like	*SlituUGT06*	6.17	7.19
111364541	UDP-glucuronosyltransferase 2B15-like	*SlituUGT07*	2.55	2.07
111364771	UDP-glucuronosyltransferase 2B19-like	*SlituUGT08*	2.21	3.79
111364777	UDP-glucuronosyltransferase 2B9-like	*SlituUGT09*	2.35	3.15
111364779	UDP-glucuronosyltransferase 2B33-like	*SlituUGT10*	2.38	4.69
111347869	UDP-glucuronosyltransferase-like	*SlituUGT11*	-2.86	-4.39
**Aminopeptidase N (APN)**				
111355387	aminopeptidase N-like	*SlituAPN06*	2.34	2.00
111357085	aminopeptidase N-like	*SlituAPN18*	-2.94	-2.10
**Serine protease (SP)**				
111351339	modular serine protease-like	*SlituSP01*	1.90	3.74
111351371	serine protease snake-like	*SlituSP02*	1.18	3.55
111351393	serine protease snake-like	*SlituSP03*	2.24	2.38
111360548	serine protease snake-like	*SlituSP04*	1.76	1.60
111362337	serine protease snake-like	*SlituSP05*	2.52	3.20
111364579	serine protease easter-like	*SlituSP06*	1.86	1.99
111347872	serine protease snake-like	*SlituSP07*	-2.36	-2.57
111351246	serine protease snake-like	*SlituSP08*	-4.93	-5.09
**Sialin**				
111354090	sialin	*SlituSialin01*	1.57	1.58
**Cuticle protein (CP)**				

111347774	cuticle protein 3-like	*SlituCP01*	3.18	3.75
111347903	larval cuticle protein LCP-17-like	*SlituCP02*	2.92	2.39
111347904	cuticle protein CP14.6-like	*SlituCP03*	4.24	3.40
111348166	pupal cuticle protein 36-like	*SlituCP04*	3.18	2.23
111348373	pupal cuticle protein 36-like	*SlituCP05*	4.41	4.02
111348459	pupal cuticle protein 36-like	*SlituCP06*	2.83	1.68
111348865	cuticle protein 16.5-like	*SlituCP07*	3.25	6.02
111348898	larval cuticle protein A2B-like	*SlituCP08*	3.94	3.12
111348899	larval cuticle protein A3A-like	*SlituCP09*	6.13	4.47
111349175	cuticle protein 8-like	*SlituCP10*	9.40	8.42
111349176	cuticle protein 7-like	*SlituCP11*	3.78	2.58
111349199	cuticle protein 7-like	*SlituCP12*	5.37	4.68
111349318	larval cuticle protein LCP-22-like	*SlituCP13*	2.91	2.61
111349454	cuticle protein 3-like	*SlituCP14*	3.20	1.93
111349551	cuticle protein 7-like	*SlituCP15*	4.14	3.24
111349696	larval cuticle protein LCP-17-like	*SlituCP16*	3.65	2.84
111349765	cuticle protein 19-like	*SlituCP17*	3.95	2.79
111349991	cuticle protein 1-like	*SlituCP18*	7.07	6.97
111350074	cuticle protein 1	*SlituCP19*	9.13	9.77
111350106	cuticle protein 16.5-like	*SlituCP20*	5.66	5.26
111350118	pupal cuticle protein PCP52-like	*SlituCP21*	5.87	5.81
111352199	larval/pupal rigid cuticle protein 66-like	*SlituCP22*	8.61	8.95
111352244	larval/pupal rigid cuticle protein 66-like	*SlituCP23*	8.78	8.37
111352245	larval/pupal rigid cuticle protein 66-like	*SlituCP24*	4.61	4.10
111352387	cuticle protein-like	*SlituCP25*	9.39	8.40
111352388	cuticle protein-like	*SlituCP26*	5.80	5.95
111355815	flexible cuticle protein 12-like	*SlituCP27*	5.10	5.41
111357474	cuticle protein 16.5-like	*SlituCP28*	1.57	2.78
111357528	cuticle protein 16.5-like	*SlituCP29*	8.09	7.39
111357529	cuticle protein 16.5-like	*SlituCP30*	6.98	7.29
111358957	larval/pupal cuticle protein H1C-like	*SlituCP31*	3.62	3.21
111358962	pupal cuticle protein C1B-like	*SlituCP32*	3.85	2.43
111358963	pupal cuticle protein C1B-like	*SlituCP33*	3.70	1.77
111358964	pupal cuticle protein C1B-like	*SlituCP34*	4.00	2.97
111359064	cuticle protein 16.5-like	*SlituCP35*	6.32	5.88
111359123	pupal cuticle protein C1B-like	*SlituCP36*	3.63	3.02
111359128	cuticle protein 16.5-like	*SlituCP37*	6.46	6.61
111359130	cuticle protein 16.5-like	*SlituCP38*	8.57	6.87
111359132	cuticle protein 38-like	*SlituCP39*	6.28	7.39
111359133	cuticle protein 65-like	*SlituCP40*	5.86	4.34
111359134	larval/pupal cuticle protein H1C-like	*SlituCP41*	4.92	4.81
111359173	cuticle protein 16.5	*SlituCP42*	3.59	2.44
111359205	pupal cuticle protein 36a-like	*SlituCP43*	4.34	5.51
111359227	larval/pupal cuticle protein H1C-like	*SlituCP44*	3.40	3.63

111359231	pupal cuticle protein C1B-like	*SlituCP45*	3.89	4.42
111359480	pupal cuticle protein 20-like	*SlituCP46*	4.89	7.39
111359684	pupal cuticle protein 20-like	*SlituCP47*	3.80	4.75
111359727	pupal cuticle protein 20-like	*SlituCP48*	4.48	4.98
111360023	larval cuticle protein LCP-17-like	*SlituCP49*	5.47	4.16
111360318	pupal cuticle protein-like	*SlituCP50*	6.90	6.76
111360374	pupal cuticle protein-like	*SlituCP51*	2.69	2.49
111362720	larval cuticle protein A3A-like	*SlituCP52*	7.25	6.56
111349704	larval cuticle protein 16/17-like	*SlituCP53*	-4.83	-5.35
111364609	larval cuticle protein A3A-like	*SlituCP54*	-5.28	-3.75
